# Response to Letter to the Editor From Bonnema et al: “Comparative Effectiveness of Levothyroxine, Desiccated Thyroid Extract, and Levothyroxine + Liothyronine in Hypothyroidism”

**DOI:** 10.1210/clinem/dgab779

**Published:** 2021-10-26

**Authors:** Mohamed K M Shakir, Daniel I Brooks, Elizabeth A McAninch, Tatiana De Lourdes Fonseca, Vinh Q Mai, Antonio C Bianco, Thanh D Hoang

**Affiliations:** 1 Walter Reed National Military Medical Center, Bethesda, Maryland 20889-5600, USA; 2 Uniformed Services University of the Health Sciences, Bethesda, Maryland 20814, USA; 3 Divsion of Endocrinology and Metabolism, Rush University Medical Center, Chicago, Illinois 60612, USA; 4 Section of Adult and Pediatric Endocrinology, University of Chicago, Chicago, Illinois 60637, USA

Dear Editor,

We thank Bonnema et al (1) for giving us the opportunity to provide further details on our recent publication (2) while addressing their insightful questions.

1. In reference to the consequences of long-term treatment with liothyronine (LT3), we respectfully point out that there is now solid data that LT3 does not increase the frequency of adverse reactions if serum thyrotropin (TSH) levels are maintained within the normal range. For example, an observational study during 1997 to 2014 (3) compared nearly 34 000 patients taking only levothyroxine (LT4) with those using LT4 + LT3 (n = 327) or LT3 alone (n = 73) during a mean follow-up of 9.3 years (SD 5.6) and a maximum follow-up of 17.3 years. The study did not reveal higher mortality or morbidity risk due to cardiovascular disease, atrial fibrillation, or fractures. In addition, an analysis of 20 clinical trials that included almost 1000 patients observed for up to 1 year indicated that peaks of serum 3,5,3′-triiodothyronine observed after the LT3 tablets only minimally affected serum TSH, heart rate, and blood pressure; the frequency of adverse reactions was similar to patients taking LT4 (4). Bone turnover markers were studied in 2 trials, and they remained within normal range. This was confirmed in a recent meta-analysis by Millan-Alanis et al (5) in which 18 clinical trials comparing LT4 vs LT4 + LT3 therapy were evaluated found no differences in adverse events. In fact, we are unaware that combination therapy containing LT3 has ever been associated with increased occurrence of adverse reactions in patients maintaining normal TSH levels.
2. In reference to a potential placebo effect associated with “entering a trial,” we found that 20 patients comprised the subgroup with the worst outcomes while on LT4 therapy and these patients were randomly distributed across the 3 treatment arms. Seven patients received LT4 in arm 1 and 9 patients received LT4 in arm 2. Thus, it is unlikely that a placebo effect played a role.
3. Baseline values were considered in the analyses. As stated under “Materials and Methods,” differences between treatments were evaluated using mixed-effects models. The primary outcome model included a fixed effect for treatment and a random effect of subject. Models were run with and without the inclusion of baseline scores to isolate between treatment differences. In the subanalysis we also considered the baseline values but were not able to detect differences between baseline and the LT4-treated arm.
4. In reference to a subgroup analysis of patients on desiccated thyroid extract (DTE) or LT4 + LT3 before the trial, we found that there were 8 patients on DTE and 3 patients on LT4/LT3 at baseline. At the end of the study, out of these 11 patients, 6 preferred DTE and 5 preferred the LT4 + LT3 combination. This suggests that a placebo effect could not explain our findings. An appropriately powered study could address this point further. Of note, a preference for DTE had already been identified in our previous study(6). It has been our experience that most patients taking DTE show a strong preference for continuing on DTE rather than switching to LT4.

## Data Availability

Some or all data generated or analyzed during this study are included in this published article or in the data repositories listed in References.

## Abbreviations

DTE: desiccated thyroid extract
LT3: liothyronine
LT4: levothyroxine
TSH: thyrotropin

## References

[1] Bonnema SJ , Riis KR, Jensen CZ, Thvilum M, Nygaard B. Letter to the editor (comparative effectiveness of levothyroxine, desiccated thyroid extract, and levothyroxine + liothyronine in hypothyroidism). J Clin Endocrinol Metab. 2021. 10.1210/clinem/dgab778 34718607

[2] Shakir KMM , Brooks DI, McAninch EA, et al. Comparative effectiveness of levothyroxine, desiccated thyroid extract, and levothyroxine + liothyronine in hypothyroidism: a subset of hypothyroid patients responds to combination therapy containing T3. J Clin Endocrinol Metab. 2021;106(11):e4400-4413. 34185829 10.1210/clinem/dgab478 PMC8530721

[3] Leese GP , Soto-Pedre E, Donnelly LA. Liothyronine use in a 17 year observational population-based study—the TEARS study. Clin Endocrinol (Oxf). 2016;85(6):918-925. 26940864 10.1111/cen.13052

[4] Idrees T , Palmer S, Maciel RMB, Bianco AC. Liothyronine and desiccated thyroid extract in the treatment of hypothyroidism. Thyroid. 2020;30(10):1399-1413. 32279609 10.1089/thy.2020.0153 PMC7640752

[5] Millan-Alanis JM , González-González JG, Flores-Rodríguez A, et al. Benefits and harms of levothyroxine/L-triiodothyronine versus levothyroxine monotherapy for adult patients with hypothyroidism: systematic review and meta-analysis. Thyroid. Published online September 8, 2021. doi:10.1089/thy.2021.0270 PMC8917901 34340589

[6] Hoang TD , Olsen CH, Mai VQ, Clyde PW, Shakir MK. Desiccated thyroid extract compared with levothyroxine in the treatment of hypothyroidism: a randomized, double-blind, crossover study. J Clin Endocrinol Metab. 2013;98(5):1982-1990. 23539727 10.1210/jc.2012-4107

